# A higher composite dietary antioxidant index is associated with a decreased risk of subclinical hyperthyroidism in adults: evidence from epidemiological studies

**DOI:** 10.3389/fnut.2025.1613223

**Published:** 2025-07-11

**Authors:** Jie Wu, Junxian Niu, Chuyu Jia, Qingkai Yang, Dewei Li, Xuqin Dong

**Affiliations:** ^1^Department of Thyroid Surgery, Shanxi Provincial People's Hospital Affiliated to Shanxi Medical University, Taiyuan, Shanxi, China; ^2^Fifth Clinical Medical College, Shanxi Medical University, Taiyuan, China; ^3^Department of Physical Examination Center, Shanxi Provincial People's Hospital Affiliated to Shanxi Medical University, Taiyuan, Shanxi, China

**Keywords:** CDAI, subclinical hyperthyroidism, thyroid diseases, oxidative stress, cross-sectional study

## Abstract

**Background and objective:**

Thyroid dysfunction is closely related to oxidative stress, and the intake of dietary antioxidants may affect thyroid function by regulating oxidative stress. The composite dietary antioxidant index (CDAI) is used as an indicator to measure the overall antioxidant capacity of the diet by quantifying the intake of these various antioxidants. However, its association with thyroid dysfunction has not been clearly established. This study aimed to investigate the association between CDAI and thyroid dysfunction in a nationally representative sample of U.S. adults.

**Methods:**

For this research, data was drawn from the National Health and Nutrition Examination Survey (NHANES) conducted in the United States between 2007 and 2012. The study population comprised 5,956 adults age ≥ 20 years. It investigated the association between CDAI and subclinical hyperthyroidism (SCHyper) as well as other thyroid dysfunctions through multivariate logistic regression and restricted cubic spline analysis (RCS).

**Results:**

After adjusting for covariates, our research revealed that an increase in CDAI is significantly associated with a reduced risk of SCHyper [OR = 0.90, 95% CI = (0.82, 0.99), *p* = 0.03]. The risk of SCHyper in the highest CDAI quartile (Q4) was reduced by 57.0% compared to the lowest group (Q1) [OR = 0.43, 95% CI = (0.20, 0.92)]. Non-linear analysis indicated an L-shaped curve relationship between CDAI and SCHyper. Subgroup analysis and interaction terms suggested that race and poverty to income ratio (PIR) subgroups affect the relationship between CDAI and incidence of SCHyper. And, the protective effect of CDAI against SCHyper was more particularly stronger in non-Hispanic whites and high-income (PIR > 3.5) populations.

**Conclusion:**

Higher CDAI scores were independently associated with a reduced risk of SCHyper, suggesting that dietary antioxidants may protect thyroid function by alleviating oxidative stress. These results offer new insights into the prevention and treatment of patients with SCHyper.

## 1 Introduction

Thyroid is one of the important glands of the endocrine system, located in the anterior inferior neck, responsible for the formation and secretion of thyroid hormones and iodine homeostasis within the human body (1). Thyroid hormones have the effect of promoting metabolism, facilitating growth and development, increasing the excitability of the nervous system, and enhancing myocardial contractility, accelerating heart rate. Thyroid hormone levels that are either above or below the normal range can cause thyroid dysfunction, which can cause damage to various body systems and lead to the development of other diseases such as kidney dysfunction (2), sleep disorders (3), and female infertility (4). Thyroid dysfunction can manifest as hypothyroidism, where the body lacks sufficient thyroid hormones, resulting in symptoms such as fatigue, weight gain, low metabolic rate, and depression (5). On the opposite end of the spectrum is hyperthyroidism, characterized by an overproduction of these hormones, which can cause symptoms like weight loss, anxiety, and increased heart rate (6).

The synthesis of thyroid hormone requires the oxidation of iodine and the catalytic activity of thyroid peroxidase, with reactive oxygen species playing a significant role in this process (7). However, an excessive accumulation of reactive oxygen species can lead to oxidative stress (OS), which is the origin of chronic diseases (8). Recent investigations demonstrate that OS is related to the pathogenesis of various diseases, including thyroid disorders (9, 10). Therefore, maintaining a balance between the production or exposure to reactive oxygen species and antioxidant defenses is crucial.

The Composite Dietary Antioxidant Index (CDAI) is a summary score that encompasses multiple dietary antioxidants, such as vitamins A, C, and E, as well as carotenoids, selenium (Se), and zinc (Zn). It reflects the overall antioxidant capacity of the diet by quantifying the intake of these various antioxidants (11). Researchers can use the CDAI to evaluate the antioxidant status of an individual’s diet and determine if it meets the recommended levels for optimal health. CDAI holds considerable utility in the prevention and control of chronic ailments. Previous studies have demonstrated that modulating antioxidant intake through diet can significantly diminish oxidative stress levels in the body (12). A study revealed that an elevated CDAI score is associated with a reduced overall likelihood of colorectal cancer development (13), and it can also reduce the risk of prostate cancer (14). Furthermore, CDAI is closely associated with numerous diseases, including depression (15), coronary heart disease (16), osteoporosis (17), and biological aging (18). Specifically in Hashimoto’s thyroiditis, OS plays a central role in pathogenesis, and antioxidant supplementation has been proposed as a potential adjuvant therapy to mitigate its deleterious effects (19). Nevertheless, the potential relationship between CDAI and the development of thyroid dysfunction has not been systematically investigated in large-scale population studies.

Consequently, this study aimed to investigate the association between CDAI and thyroid dysfunction prevalence in a nationally representative sample of U.S. adults by analyzing data from the 2007–2012 National Health and Nutrition Examination Survey (NHANES) cycles. The objective was to provide valuable insights into CDAI as a modifiable risk factor for thyroid health, which may provide assistance in the prevention and treatment of thyroid disorders.

## 2 Methods

### 2.1 Study population

The NHANES gathers crucial information regarding the incidence of significant illnesses and medical conditions, dietary habits, nutritional tendencies, and health-related behaviors across the nation through interviews and physical examinations. Administered by the National Center for Health Statistics (NCHS), which operates under the Centers for Disease Control and Prevention (CDC), all individuals involved in the study gave their informed consent before its commencement.

**Figure 1:**
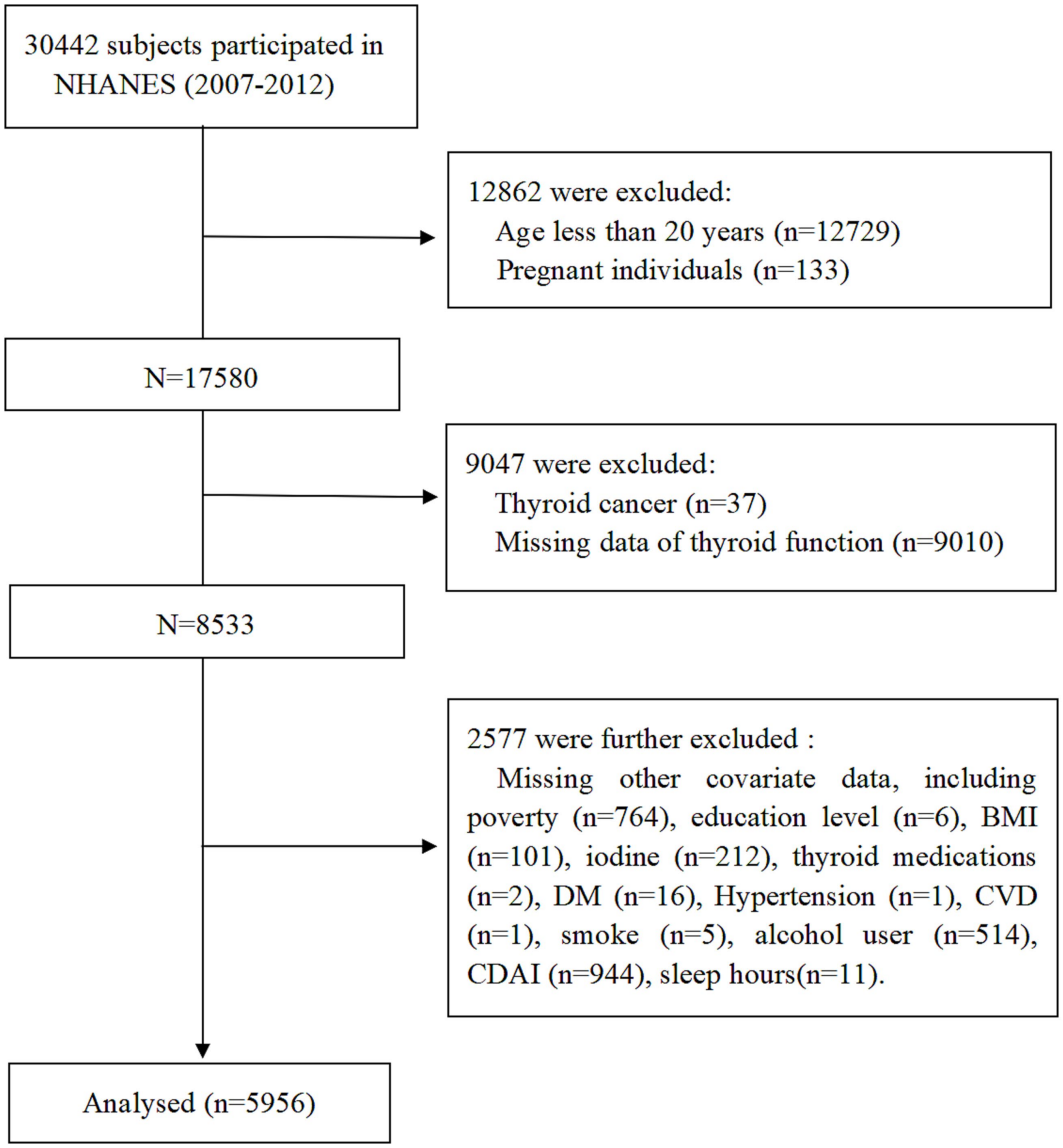
Flowchart of study population.

Data from three NHANES cycles (2007–2008, 2009–2010, and 2011–2012) involving a total of 30,442 participants. The exclusion criteria were as follows: (1) Age less than 20 years; (2) pregnant individuals; (3) history of thyroid cancer; (4) missing thyroid hormone data; (5) participants with missing covariate data; (6) missing CDAI and sleep hours data. After applying these criteria, 5,956 adults aged ≥20 years were included in the final analysis. The participant selection process is summarized in Figure 1.

### 2.2 Serum thyroid hormone and thyroid dysfunction classification

Serum thyroid function was assessed through comprehensive laboratory analyses, including measurements of thyroid-stimulating hormone (TSH), free thyroxine (FT4), free triiodothyronine (FT3), total thyroxine (TT4), total triiodothyronine (TT3), thyroid peroxidase antibodies (TPOAb), and thyroglobulin antibodies (TgAb). Reference ranges for thyroid hormones were defined as follows, based on established clinical guidelines and prior studies: TSH (0.4–4.5 mIU/L), FT4 (9–25 pmol/L), and FT3 (2.5–3.9 pg./mL) (20–22).

Normal Thyroid Function: participants with TSH within the reference range (0.4–4.5 mIU/L) and no history of thyroid hormone replacement therapy or antithyroid medication use (6). We defined thyroid dysfunction and autoimmune thyroid disease as follows: (1) Hashimoto’s thyroiditis (HT): participants with TPOAb > 9 IU/mL or TgAb > 115 IU/mL were considered positive (23). (2) Autoimmune thyroiditis (AIT): participants with TPOAb > 9 IU/mL or TgAb > 4 IU/mL were considered positive (24). (3) Subclinical hypothyroidism (SCH): TSH ≥ 4.5 mIU/L with FT4 within normal range (9–25 pmol/L), in the absence of thyroid medication use. (4) Subclinical hyperthyroidism (SCHyper): TSH < 0.4 mIU/L with FT4 (9–25 pmol/L) and FT3 (2.5–3.9 pg/mL) within normal ranges, and no current thyroid drug use. (5) Hypothyroidism: (a) participants who self-reported take medication for hypothyroidism (levothyroxine, liothyronine or desiccated thyroid) or (b) TSH ≥ 4.5 mIU/L with FT4 < 9 pmol/L, without thyroid medication use. (6) Hyperthyroidism: (a) participants who self-reported take antithyroid drugs (propylthiouracil or methimazole) or (b) TSH < 0.4 mIU/L with FT4 > 25 pmol/L or (c) TSH < 0.4 mIU/L with FT3 > 3.9 pmol/L, in the absence of thyroid medication use.

### 2.3 Composite dietary antioxidant index

The CDAI is a composite metric designed to quantify the cumulative antioxidant capacity of an individual’s diet by integrating six key dietary components: vitamins A, C, and E, as well as selenium (Se), zinc (Zn), and total carotenoids (25). Nutrient intake data were derived from two non-consecutive 24-h dietary recall assessments. The first recall was conducted in person at the Mobile Examination Center (MEC), while the second was administered via telephone 3–10 days later to minimize recall bias and capture day-to-day dietary variability.

Each micronutrient was standardized by dividing by the standard deviation (SD) after subtracting the mean. Subsequently, the CDAI score was determined by summing the standardized scores of all six components. This method ensures comparability across nutrients with differing intake ranges and units. The formula is expressed as:


$$\text{CDAI}=\sum_{i=1}^{n=6}(\text{Individual Intake}-\text{Mean})/\mathrm{SD}$$

### 2.4 Covariates

To account for potential confounding factors, covariates were selected based on prior literature and grouped into four domains: demographic, socioeconomic, dietary, and health-related variables. Demographic variables included age (stratified as 20–40, 41–60, or > 60 years), gender, and race/ethnicity (non-Hispanic White, non-Hispanic Black, Mexican American, or Other). Socioeconomic factors encompassed education level (< high school, high school graduate, or > high school) and poverty-income ratio (PIR; ≤ 1.3, 1.3–3.5, or > 3.5). Lifestyle and behavioral variables comprised smoking status (never: < 100 lifetime cigarettes; former: ≥ 100 cigarettes but abstinent; current: ≥ 100 cigarettes and actively smoking), alcohol (26, 27) (never, former, mild, moderate, or heavy), and sleep duration (< 7, 7–9, or > 9 h). Health-related covariates included body mass index (BMI: < 18.5, 18.5–24.9, 25.0–29.9, or ≥ 30 kg/m^2^), urinary iodine concentration (28) (UIC: < 100, 100–299, or ≥ 300 ug/L), and comorbidities such as hypertension, diabetes, and cardiovascular disease (CVD). All categorizations adhered to standardized clinical guidelines and prior epidemiological frameworks to ensure methodological consistency.

### 2.5 Statistical analysis

To account for the complex multistage sampling design of NHANES, all analyses incorporated appropriate sampling weights to ensure nationally representative estimates. Categorical variables were presented in the form of weighted frequencies and percentages, whereas continuous variables were described using weighted means along with their corresponding standard deviations (SD). Group comparisons were performed using the weighted chi-square test for categorical variables and weighted one-way ANOVA for continuous variables.

The relationship between CDAI and thyroid dysfunction was assessed using multivariable logistic regression analysis, structured into three hierarchical models: Model 1: unadjusted. Model 2: adjusted for age, gender, race/ethnicity, education level, sleep hours, alcohol use, smoking status, BMI, UIC, and PIR. Model 3, the fully adjusted model, was adjusted for the variables in Model 2 and further adjusted to account for additional potential confounders, such as diabetes, hypertension, and CVD. Restricted cubic spline (RCS) regression was utilized to investigate both linear and possible non-linear associations. Subgroup analyses and interaction were employed for each covariate to assess the heterogeneity in the association between CDAI and the prevalence of thyroid dysfunction. All statistical analyses were performed using R software (version 4.4.2), with statistical significance defined as *p* < 0.05 (two-tailed).

## 3 Results

### 3.1 Baseline participant characteristics

This study utilized data from the NHANES in the United States between 2007 and 2012, which included 5,956 adults aged 20 years or older. Table 1 displayed the baseline characteristics of participants stratified by CDAI quartiles. CDAI scores ranged from −8.63 to 30.61, with quartiles distributed as follows: Q1 (−8.63 to −2.28), Q2 (−2.28 to −0.12), Q3 (−0.12 to 2.47), and Q4 (2.47 to 30.61). Participants in different CDAI quartiles exhibited significant differences in age, race, education level, PIR, sleep hours, smoke, alcohol, and CVD (all *p* < 0.05). Moreover, participants in the highest quartile of CDAI demonstrated a lower prevalence of SCHyper compared with those in the lowest quartile (*p* < 0.0001). However, there were no significant differences in the prevalence of HT, AIT, SCH, hyperthyroidism, hypothyroidism, diabetes, and hypertension among the quartile groups.

**Table 1:** Baseline characteristics of participants stratified by CDAI quartiles.

Characteristics	CDAI quartiles					*p*-value
	Overall	Q1	Q2	Q3	Q4	
CDAI	[−8.63, 30.61]	[−8.63, −2.28]	(−2.28, −0.12]	(−0.12, 2.47]	(2.47, 30.61]	
Age (years)						**0.02**
20–40	1990 (38.55)^**a**^	451 (39.29)	464 (36.98)	532 (38.86)	543 (39.02)	
41–60	1977 (39.22)	474 (36.41)	485 (37.44)	477 (38.46)	541 (43.54)	
>60	1989 (22.23)	566 (24.30)	538 (25.58)	480 (22.68)	405 (17.44)	
Gender						**< 0.001**
Female	2,980 (52.33)	822 (59.80)	744 (51.32)	708 (50.01)	706 (49.77)	
Male	2,976 (47.67)	669 (40.20)	743 (48.68)	781 (49.99)	783 (50.23)	
Race/ethnicity						**0.002**
White	2,975 (72.11)	670 (68.58)	713 (67.38)	771 (74.70)	821 (76.29)	
Black	1,165 (9.91)	351 (13.09)	291 (10.65)	268 (8.79)	255 (7.95)	
Mexican	891 (7.81)	241 (7.70)	232 (10.06)	231 (7.61)	187 (6.19)	
Other race	925 (10.17)	229 (10.63)	251 (11.91)	219 (8.89)	226 (9.58)	
Education						**< 0.0001**
< High school	1,577 (17.50)	585 (28.99)	398 (17.90)	330 (14.51)	264 (11.36)	
High School	1,397 (24.04)	386 (27.62)	385 (28.60)	322 (22.71)	304 (18.77)	
> High school	2,982 (58.46)	520 (43.38)	704 (53.50)	837 (62.78)	921 (69.86)	
PIR						**< 0.0001**
≤1.3	1802 (21.02)	622 (31.45)	448 (22.02)	385 (17.10)	347 (16.04)	
1.3–3.5	2,252 (34.21)	572 (37.28)	606 (38.57)	558 (33.85)	516 (28.58)	
>3.5	1902 (44.77)	297 (31.26)	433 (39.41)	546 (49.05)	626 (55.38)	
UIC (ug/L)						0.57
<100	1885 (33.12)	446 (33.92)	498 (32.92)	463 (31.53)	478 (34.20)	
100–299	2,928 (48.70)	759 (49.57)	698 (47.18)	734 (49.09)	737 (48.96)	
≥300	1,143 (18.17)	286 (16.51)	291 (19.90)	292 (19.37)	274 (16.84)	
Sleep Hours (h)						**< 0.0001**
<7	2,368 (37.21)	667 (44.01)	617 (40.04)	551 (35.35)	533 (31.48)	
7–9	3,447 (60.84)	774 (52.57)	836 (58.36)	916 (63.55)	921 (66.58)	
>9	141 (1.94)	50 (3.41)	34 (1.60)	22 (1.10)	35 (1.94)	
Smoke						**< 0.0001**
Never	3,145 (53.68)	682 (43.76)	779 (50.67)	830 (56.37)	854 (61.12)	
Former	1,593 (24.92)	387 (22.26)	402 (25.06)	397 (26.37)	407 (25.42)	
Now	1,218 (21.40)	422 (33.99)	306 (24.27)	262 (17.27)	228 (13.46)	
BMI (kg/m^2^)						**0.05**
<18.5	86 (1.34)	32 (1.62)	20 (1.02)	15 (1.28)	19 (1.44)	
18.5–24.9	1,585 (29.58)	360 (27.80)	351 (25.35)	408 (29.70)	466 (34.37)	
25.0–29.9	2054 (33.78)	526 (34.40)	537 (33.91)	522 (35.20)	469 (31.87)	
≥30.0	2,231 (35.30)	573 (36.18)	579 (39.71)	544 (33.82)	535 (32.32)	
Alcohol						**< 0.0001**
Never	817 (10.31)	285 (14.73)	197 (11.57)	169 (8.66)	166 (7.48)	
Former	1,173 (16.53)	358 (21.00)	337 (18.74)	250 (13.88)	228 (13.82)	
Mild	1898 (33.65)	353 (24.19)	455 (30.03)	539 (36.20)	551 (41.39)	
Moderate	897 (17.49)	194 (14.78)	210 (16.28)	243 (20.29)	250 (17.89)	
Heavy	1,171 (22.02)	301 (25.30)	288 (23.38)	288 (20.96)	294 (19.43)	
HT						0.31
Yes	698 (12.40)	168 (11.40)	181 (11.86)	177 (11.97)	172 (14.00)	
No	5,258 (87.60)	1,323 (88.60)	1,306 (88.14)	1,312 (88.03)	1,317 (86.00)	
AIT						0.63
Yes	859 (15.54)	210 (14.57)	213 (14.66)	226 (16.07)	210 (16.50)	
No	5,097 (84.46)	1,281 (85.43)	1,274 (85.34)	1,263 (83.93)	1,279 (83.50)	
Hyperthyroidism						0.30
Yes	17 (0.16)	5 (0.28)	4 (0.16)	3 (0.06)	5 (0.17)	
No	5,939 (99.84)	1,486 (99.72)	1,483 (99.84)	1,486 (99.94)	1,484 (99.83)	
SCHyper						**< 0.0001**
Yes	88 (1.30)	28 (3.01)	23 (0.83)	20 (0.71)	17 (0.97)	
No	5,868 (98.70)	1,463 (96.99)	1,464 (99.17)	1,469 (99.29)	1,472 (99.03)	
SCH						0.51
Yes	148 (2.86)	46 (3.71)	35 (2.96)	35 (2.24)	32 (2.70)	
No	5,808 (97.14)	1,445 (96.29)	1,452 (97.04)	1,454 (97.76)	1,457 (97.30)	
Hypothyroidism						0.67
Yes	456 (8.37)	105 (7.95)	118 (7.63)	123 (8.95)	110 (8.77)	
No	5,500 (91.63)	1,386 (92.05)	1,369 (92.37)	1,366 (91.05)	1,379 (91.23)	
Hypertension						0.16
Yes	2,558 (35.64)	711 (37.46)	676 (38.66)	607 (33.81)	564 (33.46)	
No	3,398 (64.36)	780 (62.54)	811 (61.34)	882 (66.19)	925 (66.54)	
Diabetes						0.07
Yes	1,136 (13.59)	327 (13.61)	312 (16.20)	273 (13.41)	224 (11.55)	
No	4,820 (86.41)	1,164 (86.39)	1,175 (83.80)	1,216 (86.59)	1,265 (88.45)	
CVD						**< 0.0001**
Yes	683 (8.05)	225 (10.17)	186 (9.71)	154 (8.50)	118 (4.62)	
No	5,273 (91.95)	1,266 (89.83)	1,301 (90.29)	1,335 (91.50)	1,371 (95.38)	

^**a**^Actual frequencies (weighted percentages).

CDAI, composite dietary antioxidant index; PIR, poverty to income ratio; UIC, urinary iodine concentration; BMI, body mass index; HT, Hashimoto’s thyroiditis; AIT, autoimmune thyroiditis; SCHper, subclinical hyperthyroidism; SCH, subclinical hypothyroidism; CVD, cardiovascular disease. Bold values (*p* < 0.05) indicate statistically significant associations.

### 3.2 Association between CDAI and SCHyper

The association between CDAI and SCHyper was presented in Table 2. When analyzed as a continuous variable, CDAI consistently demonstrated a negative association with SCHyper risk across all regression models [Model 1: OR = 0.87, 95% CI = (0.80, 0.94), *p* < 0.001; Model 2: OR = 0.90, 95% CI = (0.82, 0.99), *p* = 0.03; Model 3: OR = 0.90, 95% CI = (0.82, 0.99), *p* = 0.03]. In the fully adjusted model, each 1-unit increase in CDAI was associated with a 10.0% reduction in SCHyper prevalence. Furthermore, when compared to the lowest quartile (Q1) of CDAI levels, the second (Q2), third (Q3), and highest quartiles (Q4) also exhibited a negative correlation with the risk of SCHyper [Q2: OR = 0.30, 95% CI = (0.16, 0.58), *p =* 0.001; Q3: OR = 0.29, 95% CI = (0.12, 0.72), *p* = 0.01; Q4: OR = 0.43, 95% CI = (0.20, 0.92), *p =* 0.03; respectively]. And the negative trend remained statistically significant (*P* for trend = 0.01).

**Table 2:** Association between CDAI and SCHyper.

Variables	Model 1		Model 2		Model 3	
	OR (95%CI)	*p*	OR (95%CI)	*p*	OR (95%CI)	*p*
CDAI	0.87 (0.80, 0.94)	**<0.001**	0.90 (0.82, 0.99)	**0.03**	0.90 (0.82, 0.99)	**0.03**
Categories						
Q1	ref		ref		ref	
Q2	0.27 (0.14, 0.51)	**<0.001**	0.30 (0.16, 0.57)	**<0.001**	0.30 (0.16, 0.58)	**0.001**
Q3	0.23 (0.11, 0.49)	**<0.001**	0.29 (0.12, 0.70)	**0.01**	0.29 (0.12, 0.72)	**0.01**
Q4	0.32 (0.16, 0.63)	**0.002**	0.42 (0.19, 0.93)	**0.03**	0.43 (0.20, 0.92)	**0.03**
p for trend		**<0.001**		**0.005**		**0.01**

Model 1: unadjusted for any covariates.

Model 2: adjusted for age, gender, race/ethnicity, education level, sleep hours, alcohol, smoking status, BMI, UIC, and PIR.

Model 3: the fully adjusted model, was adjusted for the variables in Model 2 and further adjusted to account for additional potential confounders, such as diabetes, hypertension, and CVD.

CDAI, composite dietary antioxidant index; SCHyper, subclinical hyperthyroidis; OR, odds ratio; CI, confidence interval; ref, reference; UIC, urinary iodine concentration; PIR, poverty to income ratio; BMI, body mass index; CVD, cardiovascular disease. Bold values (*p* < 0.05) indicate statistically significant associations.

**Figure 2:**
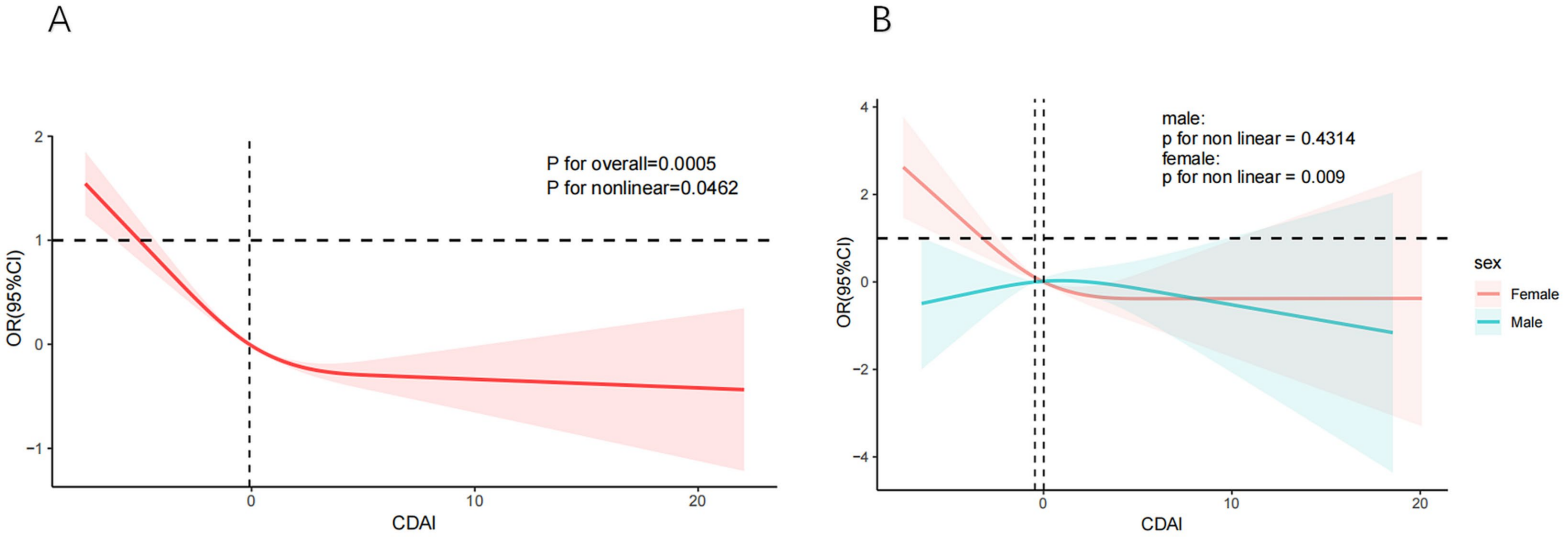
The non-linear relationships between CDAI and SCHyper. Models adjust age, gender, race/ethnicity, education level, sleep hours, alcohol, smoking status, BMI, UIC, PIR, diabetes, hypertension, and CVD. **(A)** CDAI and SCHyper in adults; **(B)** CDAI and SCHyper by gender. CDAI, composite dietary antioxidant index; OR, odds ratio; CI, confidence interval; PIR, poverty to income ratio; UIC, urinary iodine concentration; BMI, body mass index; CVD, cardiovascular disease.

The RCS curves were employed to evaluate both linear and potential non-linear associations between CDAI and SCHyper. The relationship between CDAI and SCHyper displayed a clear L-shaped curve pattern among all participants (*P* for non-linearity = 0.0462, as shown in Figure 2A) and in female adults (*P* for non-linearity = 0.009, as shown in Figure 2B). The curve demonstrated a steeper decline in SCHyper risk at lower CDAI levels, with the protective effect plateauing as CDAI increased further.

### 3.3 Association of individual antioxidant components with SCHyper

Considering that the data for individual antioxidant intake (vitamins A, C, E, Se, Zn, and carotenoids) does not follow a normal distribution, logarithmic transformations were applied to normalize their distributions. The transformed variables (denoted as lnVa, lnVc, lnVe, lnSe, lnZn, and lnCar) were validated for normality using Shapiro–Wilk tests. The relationship between individual dietary antioxidants and SCHyper was presented in Table 3.

**Table 3:** Association between individual dietary antioxidant components and SCHyper.

Variables	Model 1		Model 2		Model 3	
	OR (95% CI)	*p*	OR (95% CI)	*p*	OR (95% CI)	*p*
lnVa	0.38 (0.19, 0.78)	**0.01**	0.59 (0.22, 1.56)	0.27	0.60 (0.22, 1.64)	0.31
lnVc	0.54 (0.32, 0.93)	**0.03**	0.61 (0.32, 1.18)	0.14	0.60 (0.31, 1.17)	0.13
lnVe	0.14 (0.06, 0.32)	**<0.0001**	0.25 (0.06, 1.10)	0.07	0.24 (0.05, 1.16)	0.07
lnZn	0.11 (0.03, 0.49)	**0.004**	0.23 (0.05, 1.09)	0.06	0.24 (0.05, 1.05)	0.06
lnSe	0.16 (0.05, 0.57)	**0.01**	0.31 (0.09, 1.15)	0.08	0.33 (0.09, 1.21)	0.09
lnCar	0.56 (0.38, 0.83)	**0.004**	0.67 (0.40, 1.14)	0.13	0.68 (0.40, 1.16)	0.15

Model 1: unadjusted for any covariates.

Model 2: adjusted for age, gender, race/ethnicity, education level, sleep hours, alcohol, smoking status, BMI, UIC, and PIR.

Model 3: the fully adjusted model, was adjusted for the variables in Model 2 and further adjusted to account for additional potential confounders, such as diabetes, hypertension, and CVD.

SCHyper, subclinical hyperthyroidis; OR, odds ratio; CI, confidence interval; Va, vitamin A; Vc, vitamin C; Ve, vitamin E; Zn, zinc; Se, selenium; Car, carotenoids; UIC, urinary iodine concentration; PIR, poverty to income ratio; BMI, body mass index; CVD, cardiovascular disease. Bold values (*p* < 0.05) indicate statistically significant associations.

In unadjusted models (Model 1), all six antioxidants exhibited inverse associations with SCHyper risk (*p* < 0.05 for all). However, after full adjustment for covariates, none retained statistical significance (*p* > 0.05 for all).

**Figure 3:**
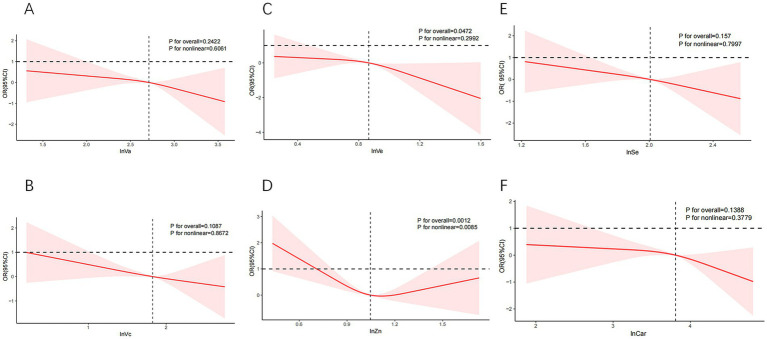
The curve relationships between individual dietary antioxidant components and SCHyper. Models adjust age, gender, race/ethnicity, education level, sleep hours, alcohol, smoking status, BMI, UIC, PIR, diabetes, hypertension, and CVD. **(A)** lnVa and SCHyper; **(B)** lnVc and SCHyper; **(C)** lnVe and SCHyper; **(D)** lnZn and SCHyper; **(E)** lnSe and SCHyper in adults; **(F)** lnCar and SCHyper. Va, vitamin A; Vc, vitamin C; Ve, vitamin E; Zn, zinc; Se, selenium; Car, carotenoids; OR, odds ratio; CI, confidence interval; PIR, poverty to income ratio; UIC, urinary iodine concentration; BMI, body mass index; CVD, cardiovascular disease.

As shown in Figure 3, we observed a U-shaped curve association between dietary Zn and SCHyper (*P* for nonlinear = 0.0085), with both deficient and excessive Zn levels associated with elevated SCHyper prevalence. In contrast, no significant nonlinear relationship was detected for the remaining antioxidant nutrients (all *P*-nonlinear > 0.05) in this dose–response analysis.

### 3.4 Subgroup analysis

**Figure 4:**
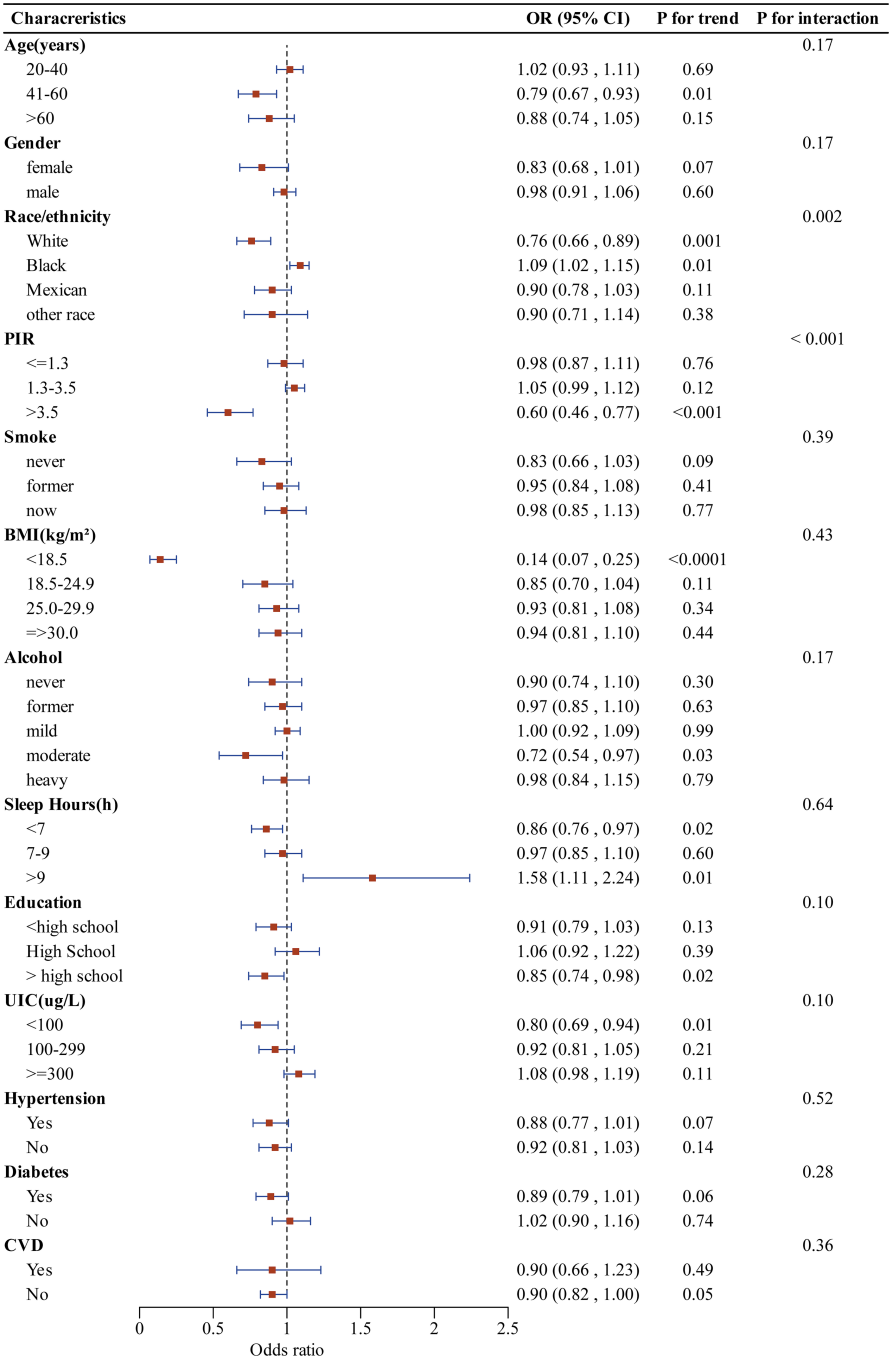
Forest plot illustrating the relationship between CDAI and SCHyper risk within each subgroup. Data are presented as OR (95%CI). Each subgroup adjusted for all factors (age, gender, race/ethnicity, education level, sleep hours, alcohol, smoking status, BMI, UIC, PIR, diabetes, hypertension, and CVD) except the stratification factor itself. CDAI, composite dietary antioxidant index; OR, odds ratio; CI, confidence interval; PIR, poverty to income ratio; UIC, urinary iodine concentration; BMI, body mass index; CVD, cardiovascular disease.

The subgroup analysis of selected covariates, using multivariate logistic regression and interaction tests, was aimed to assess the heterogeneity of the relationship between CDAI and SCHyper risk. In Figure 4, we observed significant interactions in race (*P* for interaction = 0.002) and PIR (*P* for interaction < 0.001). More precisely, the stratified subgroup analysis of covariates revealed that the protective impact of CDAI against SCHyper was more marked within the population aged between 41 and 60 years, the non-Hispanic white subgroup, the subgroup with a PIR greater than 3.5, the subgroup with BMI below 18.5, the subgroup of moderate alcohol users, the subgroup with sleep hours less than 7, and the subgroup with education levels more than high school (*p* < 0.05 for all).

## 4 Discussion

Our study is a cross-sectional investigation into the association between the CDAI and the prevalence of thyroid dysfunction among adults in the United States. The research indicated that higher CDAI are associated with a decreased risk of SCHyper. A subgroup analysis and interaction tests was conducted, revealing that the protective effect of CDAI against SCHyper was influenced by race and PIR. Additionally, RCS revealed a nonlinear L-shaped relationship between CDAI and SCHyper.

Previous studies have well-documented the association between individual antioxidants and thyroid function. A Polish team has shown in numerous studies that patients with HT, even in the euthyroid phase, have both brain bioelectrical dysfunction and changes in brain metabolism as found in neuroimaging studies. These are most likely the cause of numerous non-endocrine manifestations of the disease and prove that euthyroidism is not an indicator of the body’s balance (29, 30). For this reason, it is important to look for methods to support treatment, such as by supplementing with antioxidants, adjusting one’s diet, and altering lifestyle habits to alleviate the adverse impact of oxidative stress on the thyroid, improving thyroid function and related symptoms (31).

Selenium (Se) plays a crucial role in the metabolic processes of thyroid hormones and provides assistance in counteracting oxidative stress (32). When Se binds with selenoproteins, it can enhance the defense of thyroid cells against reactive oxygen species (ROS) (33). Vitamin A has beneficial effects on thyroid function, whether it is acting independently or in combination with iodized salt supplementation (34). Furthermore, vitamin A may be related to the metabolism of thyroid hormones in peripheral tissues. Studies on mice have found (35) that a deficiency in vitamin A reduces the hepatic conversion of T4 to T3 and decreases tissue uptake of T3, thereby increasing T4 and T3 levels. The research by Jubiz (36) indicates that for patients with hypothyroidism, vitamin C can raise serum FT4 and FT3 levels and decrease TSH. And, vitamin C can effectively reduce circulating TPOAb titers in patients with autoimmune thyroiditis (37). Vitamin E can reduce the prevalence of autoimmune thyroiditis in men (38). However, a higher dietary intake of Zn is positively correlated with an increased risk of autoimmune thyroiditis and serves as an independent risk factor. High levels of Zn can induce oxidative damage and mitochondrial changes in neurons, as well as severely impair neutrophil phagocytosis and T-cell function, thereby affecting the normal regulation of immune responses and increasing the risk of autoimmune diseases (39).

Our studies have shown that CDAI consistently exhibited a negative correlation with SCHyper risk, and the dose–response relationship follows an L-shaped curve, suggesting that the protective effect of antioxidants stabilizes after reaching a certain threshold. Furthermore, upon examining the individual effects of the six dietary antioxidants on SCHyper, we discovered that they were negatively correlated with the risk of SCHyper only in the unadjusted Model 1. While individual antioxidants, such as zinc, exhibit a U-shaped relationship, the composite design of the CDAI mitigates these risks through synergistic interactions. Research indicates that co- supplementation with Zn and Se synergistically increases the levels of antioxidant stress markers, such as glutathione peroxidase and superoxide dismutase (40). Similarly, vitamins C and E demonstrate a synergistic effect in combating lipid peroxidation, as vitamin C can repair tocopherol free radicals associated with vitamin E (41). Furthermore, studies show low Se and Vd levels in new hyperthyroidism patients, and supplementation alongside treatment aids early control (42). Simultaneous Zn and Se intake can also significantly alter thyroid hormone levels (increasing FT3 and FT4, decreasing TSH) (43). These findings underscore the complexity of antioxidant-thyroid interactions and suggest that the protective effect of CDAI on SCHyper arises from synergistic interactions among antioxidants rather than isolated components. Investigations focusing on isolated antioxidants have inherent limitations, primarily failing to account for potential synergistic or antagonistic interactions between micronutrients. Moreover, it is also unrealistic to ingest only a single antioxidant in daily diets, as foods are typically rich in a variety of nutrients. Consequently, compared to a single antioxidant, CDAI serves as an indicator that not only reflects the overall level of dietary antioxidant capacity but may also overcome the limitations of individual nutrients through the complementary effects of its components.

In the subgroup analysis and interaction terms of this study, it was found that race and PIR subgroups may affect the relationship between CDAI and incidence of SCHyper. Moreover, the protective effect of CDAI against SCHyper was more pronounced in the non-Hispanic white subgroup and among high-income individuals (PIR > 3.5). Previous studies have demonstrated that socioeconomic conditions are significantly associated with the prevalence of thyroid disorders, with incidences of both hypothyroidism and hyperthyroidism exhibiting a clear upward trend as income levels decrease (44). Educational and income disparities contribute to variations in nutritional intake across different racial groups. This dietary difference between races may be one of the main factors contributing to the variation in rates of thyroid dysfunction. Furthermore, prior research has indicated that black race, females, and older individuals were risk factors for hyperthyroidism (45).

Globally, iodine deficiency, aside from the administration of levothyroxine, is the primary risk factor for subclinical hyperthyroidism (46). Iodine functions as an antioxidant, inhibiting the formation of free radicals and ROS. Iodine deficiency can lead to an elevated incidence of thyroid nodules, which is subsequently associated with an increase in hyperthyroidism cases (47–49). Meanwhile, there were no significant interactions between the CDAI and other relevant risk factors, indicating that no additional factors were identified that influenced the association between the CDAI and SCHyper. These findings support the integration of the CDAI into personalized preventive strategies for thyroid disorders. This emphasizes tailored nutritional interventions based on gender, race, economic status, and biomarker profiles. Specifically, antioxidant-rich diets are recommended for low-income, low-education, and non-Hispanic black groups to optimize health outcomes.

Although our research has achieved certain results, it must also be acknowledged that there are some limitations. Firstly, since the data comes from the NHANES database, which employs a cross-sectional design, it is not possible to determine the causal relationship between CDAI and SCHyper. Secondly, dietary recall data are susceptible to measurement bias, although NHANES’s rigorous protocol minimizes this concern. Thirdly, our current study design was concentrated on dietary antioxidant intake and did not evaluate the levels of pro-oxidant markers. Finally, despite our exhaustive efforts to eliminate potential confounding variables, there remain certain elements beyond our complete control that may impact the ultimate result.

## 5 Conclusion

Our study indicates that higher scores of CDAI are significantly associated with a decreased risk of SCHyper, suggesting that consuming a comprehensive array of dietary antioxidants may offer protection for thyroid health. Further prospective cohort studies and mechanistic investigations will help clarify the clinical application value of CDAI in the prevention of thyroid diseases.

## Data Availability

The datasets presented in this study can be found in online repositories. The names of the repository/repositories and accession number(s) can be found at: https://www.cdc.gov/nchs/nhanes/.
